# Phosphate Cements Based on Calcined Dolomite: Influence of Calcination Temperature and Silica Addition

**DOI:** 10.3390/ma14143838

**Published:** 2021-07-09

**Authors:** Cristina Andreea Vijan, Alina Badanoiu, Georgeta Voicu, Adrian Ionut Nicoara

**Affiliations:** Department of Science and Engineering of Oxide Materials and Nanomaterials, Faculty of Applied Chemistry and Materials Science, University Politehnica of Bucharest, 1-7 Gheorghe Polizu Street, 011061 Bucharest, Romania; cris_deea_vajan@yahoo.com (C.A.V.); georgeta.voicu@upb.ro (G.V.); adrian.nicoara@upb.ro (A.I.N.)

**Keywords:** phosphate cements, calcined dolomite, quartz sand, calcined magnesite, temperature, K-struvite, industrial waste, chromium

## Abstract

The aim of this study is to assess the possibility of obtaining phosphate cements based on dolomite calcined at various temperatures with/without quartz sand addition. A lower calcination temperature of dolomite (1200 °C) determines a high increase in the system temperature when calcined dolomite is mixed with KH_2_PO_4_ (MKP) solution and also a rapid expansion of the paste. The increase in calcination temperature up to 1400 °C reduces the oxides reactivity; however, for lower dosages of MKP, the expansion phenomenon is still recorded. The increase in MKP dosage increases the compressive strength due to the formation of K-struvite. The mixing of dolomite with sand, followed by thermal treatment at 1200 °C, modifies its composition and reactivity; the compressive strength of phosphate cements obtained by mixing this solid precursor with MKP increases up to 28 days of curing. We assessed the nature of hydrates formed in the phosphate systems studied by X-ray diffraction in order to explain the hardening processes and the mechanical properties of these systems. The microstructure and elemental composition of hardened cement pastes were assessed by scanning electronic microscopy with energy-dispersive spectroscopy. The phosphate cements based on calcined magnesite or dolomite were used to immobilize an industrial hazardous waste with high chromium content. The partial substitution of calcined magnesite/dolomite with this waste determines an important decrease in compressive strengths. Nevertheless, the leaching tests confirm an adequate immobilization of chromium in some of the matrices studied (for a waste dosage corresponding to 0.5 wt % Cr).

## 1. Introduction

Magnesium phosphate cement (MPC) hardens due to an acid–base reaction between magnesia (MgO) and phosphate acid or a phosphate salt solution [1,2,3,4]. The usual source of magnesium oxide is magnesite (magnesium carbonate), which is thermally treated at increasing temperatures to obtain caustic calcined magnesite/magnesia (CCM), dead burned magnesite/magnesia (DBM), and fused magnesia (FM). CCM has numerous applications such as hydrometallurgy, steel industry, ceramic and cement manufacture, fertilizers, water treatment, etc. [5,6]. DBM and FM are mainly used in the manufacturing process of refractory materials [5], but DBM is also a key ingredient in MPC manufacture.

In the 2014 “Report on critical raw materials for EU”, magnesite was identified as a critical raw material [5]. Taking into account this aspect, it is important to find alternative sources of raw materials for the manufacture of MPC. 

Dolomite is a sedimentary rock containing calcium and magnesium carbonates [7,8]. Dolomite is an important material in various industries such as the pharmaceutical industry, metallurgy, the production of paper, inorganic binders, concrete, fertilizer, refractory bricks, water treatment, absorption of heavy metals, etc. [8,9,10]. 

The calcination of dolomite is used to transform this mineral into magnesium and calcium oxides. Depending on the experimental conditions, i.e., chemical composition of dolomite, presence and amount of impurities, grain size distribution, decomposition temperature and atmosphere (air, carbon dioxide, nitrogen etc.), the thermal decomposition of dolomite in MgO and CaO can proceed in one or several steps (endothermic processes) [8,11,12,13]. 

Yu et al. [14] studied the possibility of using dolomite as raw material to produce magnesium phosphate cement. According to these authors, mixing fine dolomite with coarse quartz sand and thermal treatment at relatively low temperatures (1100–1250 °C) substantially reduces the amount of free lime, and the MgO obtained has an adequate reactivity vs. phosphate salt (NH_4_H_2_PO_4_). The compressive strengths of phosphate cements pastes prepared with this type of calcined dolomite can reach 22 MPa after 3 h of hardening and 63 MPa after 7 days, with sufficient soundness [14]. 

MPCs can be used for the immobilization of various types of wastes with heavy metals content such as Ni, Pb, Cr, Cd, etc. [15,16,17,18,19]. Heavy metals are toxic and can cause serious health problems. Chromium (especially Cr (VI)) has an important toxic effect; it can produce skin irritation and ulcerations as well as liver and kidney deficiency and, if inhaled, it increases lung cancer risk [20].

Deng et al. [16] studied the influence of Cr^3+^ (brought in the system by Cr(NO_3_)_3_·9H_2_O) on the compressive strength, microstructure, as well as leaching toxicity of solidified forms into MPCs based on calcined magnesite and KH_2_PO_4_. According to these authors, the presence of Cr^3+^ changed the system’s pH and affected the morphology of hydration products; however, the MPCs leaching toxicity was less than the one assessed for other matrices i.e., geopolymer, calcium aluminum cement, and alkali-activated slag binders.

Therefore, we assessed in this paper the possibility of producing phosphate cements by replacing calcined magnesite with dolomite thermally treated at various temperatures, with/without quartz sand addition. We also evaluated the efficiency of magnesium phosphate cements (MPC) based on calcined magnesite and magnesium and calcium phosphate cements (MCPC) based on calcined dolomite in order to immobilize an industrial waste with high chromium content. To the best of our knowledge, the immobilization of chromium in phosphate cements based on calcined dolomite has been first reported in this paper.

## 2. Materials and Methods

The precursors used in this study were as follows:

- Calcined magnesite (M), industrial product (Tremag, Tulcea, Romania), obtained by the calcination of magnesite at 1500 °C; the residue on sieve 90 microns mesh was 7.93%.
- Calcined dolomites, obtained by thermal treatment of natural dolomite (Rodbungrup, Bucharest, Romania) at 1200 °C (D_12_) and 1400 °C (D_14_) for 3 h. The natural dolomite had a content of 47% CaCO_3_ and 37.5% MgCO_3_ and a residue on 90 microns mesh of 24.83%. After the thermal treatment, the calcined dolomites were ground up to a fineness corresponding to total passing through a 90 microns sieve.
- Calcined mixture of dolomite and quartz sand (D_12S_); the sand (Societe Nouvelle du Litoral, Leucate, France) had a fineness corresponding to total passing through a 200 microns sieve. The dolomite to quartz sand ratio was 1.5, and the thermal treatment was performed at 1200 °C for 1 h, based on the results reported by Yu et.al. [14]. The rate of heating was 10 °C/minute, and the cooling was performed in the oven.
- Potassium dihydrogen phosphate (KH_2_PO_4_—MKP), chemical reagent Sigma-Aldrich (Darmstadt, Germany).
- Setting retarder—borax (B)—chemical reagent Sigma-Aldrich (Darmstadt, Germany).
- Industrial waste with high chromium content (2.71%) in the form of CaCrO_4_.2H_2_O [21]; CaCO_3_, Mg(OH)_2_, and Ca(OH)_2_ were also detected by X-ray diffraction in this waste. The waste also contains Si, Al, Fe, and S as well as very small amounts of As, Ba, Cu, Hg, Mn, V, W, Zn and Zr [21].

The compositions of the magnesium (and calcium) phosphate cements are presented in Table 1.

The specimens were obtained by the mixing of solid component (calcined magnesite or calcined dolomite) with potassium dihydrogen phosphate, water, and in some cases borax; the resulting paste was poured in rectangular molds (15 mm × 15 mm × 60 mm- width × heigh × length). The curing of specimens was performed in the mold the first 24 h and then, after demolding, in air at 20 ± 2 °C. 

The reactive CaO and MgO content (available for the reaction with water) of calcined dolomite was determined according to the method presented in the standard SR EN 459-2 [22]. The available (unbound) CaO and MgO and corresponding hydroxides are dissolved in a sucrose solution and titrated with hydrochloric acid.

A Shimadzu XRD 6000 (Shimadzu, Kyoto, Japan), CuKα (λ = 1.5406 Å), 2*θ* ranging between 10 and 60, a 0.02 step size, and a 2 deg./min scan speed was used for X ray diffraction analyses.

The microstructure of pastes was assessed by Scanning Electron Microscopy (SEM) using an FEI Inspect F50 (Thermo Fisher—former FEI, Eindhoven, Nederland) electronic microscope equipped with a Schottky emission electron beam with a resolution of 1.2 nm at 30 kV and 3 nm at 1 kV (BSE). In this analysis, the freshly fractured samples were visualized in a vacuum mode using a 30 kV acceleration voltage and spot 3.5.

A differential thermal analyzer Shimadzu DTG-TA 51H (Shimadzu, Kyoto, Japan) was used for complex thermal analysis (DTA-TG); the analyses were performed in air, with a heating rate of 10 °C/minute, in the temperature range 20–1000 °C.

Prismatic specimens (15 mm× 15 mm × 60 mm- width × heigh × length), cured for 1 up to 28 days in air at 20 ± 2 °C, were employed for the assessment of compressive strength using a Matest testing machine (Matest, Treviolo, Italy). For the calculation of average compressive strength, a minimum of 6 compressive strength values were considered. The outliers (±10%) were not considered in calculation.

The chromium leaching test was performed according to the method presented in standard SR EN 12457-4: 2003 [23]. The MPC and MCPC specimens, hardened in air at 20 ± 2 °C for 28 days, were triturated and sieved; the particles smaller than 10 mm were mixed with water (water to solid ratio was 10). The resulting suspension was stirred for 24 h at a rate of 10 rpm by means of an orbital shaker (Heidolph Instrument Gmbh&Co.KG, Schwabach, Germany); next, the suspension was filtered, and the leachate was mixed with nitric acid to achieve a pH lower than 2. An atomic absorption spectrometer (Analytik, Jena, Germany) was used to assess the heavy metals concentration in the leachate.

## 3. Results

**Figure 1:**
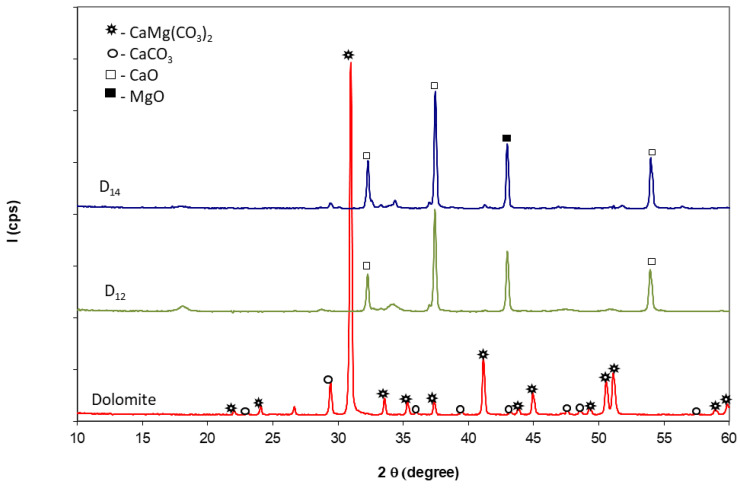
XRD patterns of dolomite and dolomite calcined at 1200 °C (D_12_) and 1400 °C (D_14_) for 3 h.

The X-ray diffraction patterns of natural dolomite presented in Figure 1 confirm the presence of dolomite (CaMg(CO_3_)_2_) along with a small amount of calcite (CaCO_3_).

**Figure 2:**
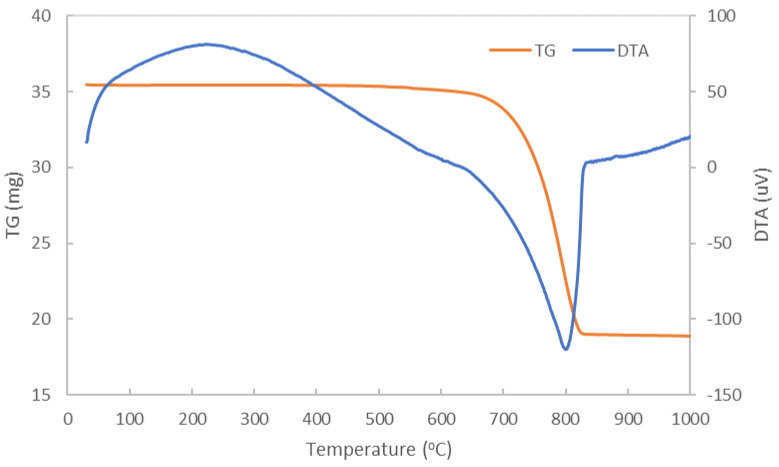
TG-DTA curves of dolomite.

The complex thermal analysis of dolomite (Figure 2) shows on the DTA curve an endothermic process with a shoulder at approximately 650 °C and maximum at 800 °C with a corresponding weight loss of 46.35% (assessed on TG curve). This process, which ends at 850 °C, can be attributed to the decarbonation of the magnesium carbonate and the calcium carbonate with CaO and MgO formation [8,11,12,13].

In correlation with previous results, the thermal treatment of dolomite at 1200 °C and 1400 °C leads to the transformation of calcium magnesium carbonate (083-1530) and calcium carbonate (072-1652) into magnesium oxide (004-0829) and calcium oxide (082-1690) (Figure 1). There are no significant differences between the XRD patterns of dolomite thermally treated at these two temperatures.

**Figure 3:**
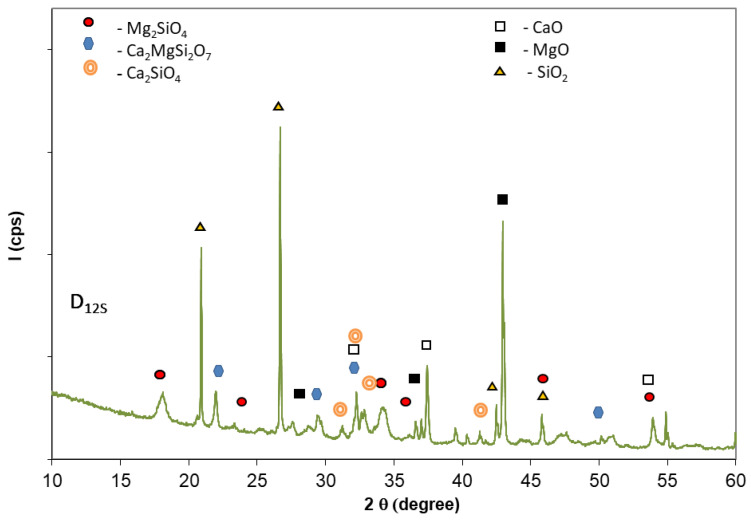
XRD patterns of dolomite with sand calcined at 1200 °C (D_12S_) for 1 h.

The mixing of dolomite with quartz sand and thermal treatment at 1200 °C for 1 h determines, as expected, the formation of calcium and/or magnesium silicates (see Figure 3). The XRD patterns presented in Figure 3 also show the presence of SiO_2_ along with MgO. The intensities of XRD peaks of CaO are much smaller (as compared with those assessed on D_12_ and D_14_ XRD patterns—Figure 1) due to its partial consumption in the reaction with SiO_2_. 

The amount of reactive calcium and magnesium oxides assessed by the method presented in SR EN 459-2 [22] in the dolomite thermally treated for 3 h at 1200 °C was 54.13%, and for the dolomite calcined at 1400 °C, it was 22%. The decrease of oxides’ reactivity when the thermal temperature increases is due, as in the case of thermal treatment of limestone, to the increase of oxides’ crystals sizes correlated with the decrease of porosity, when the material is thermally treated at a higher temperature [24,25,26,27]. 

The mixing with water (W) or KH_2_PO_4_ solution (MKP) determines, for the specimens based on the dolomite calcined at 1200 °C, an intense and rapid heat release (Table 2), due to the hydration of MgO and CaO (in the case of D_12__W) and to the reaction with the MKP (in the case of D_12__MKP_4 and D_12__MKP_2.5). It has been noticed that a decrease of MKP content determines a slower heat release corresponding to the exothermic processes specific for the setting and hardening of these phosphate systems i.e., the maximum temperature assessed on pastes is reached after a longer time (see D_12__MKP_4 as compared to D_12__MKP_2.5—Table 2).

**Figure 4:**
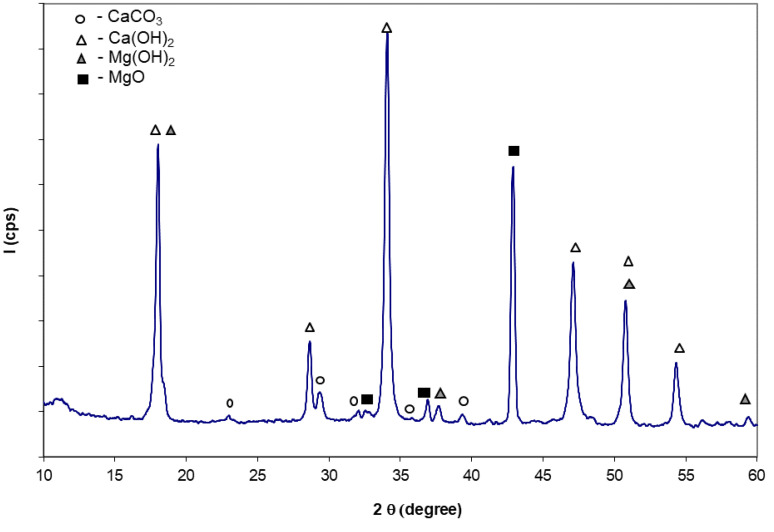
XRD patterns of paste obtained by mixing of water with dolomite calcined at 1200 °C/3 h (D_12__W).

Figure 4 shows the XRD patterns of sample D_12__W after 3 days of hardening. It can be observed the presence of Ca(OH)_2_ (084-1276) resulting from the hydration of CaO and the presence of Mg(OH)_2_ (084-2163) resulting from the hydration of MgO—both exothermic processes that explain the significant temperature increase; the presence of MgO peaks on the XRD patterns confirms the smaller reactivity vs. water of this oxide as compared to CaO [24,27,28], which correlates with the thermal treatment temperature and plateau. 

**Figure 5:**
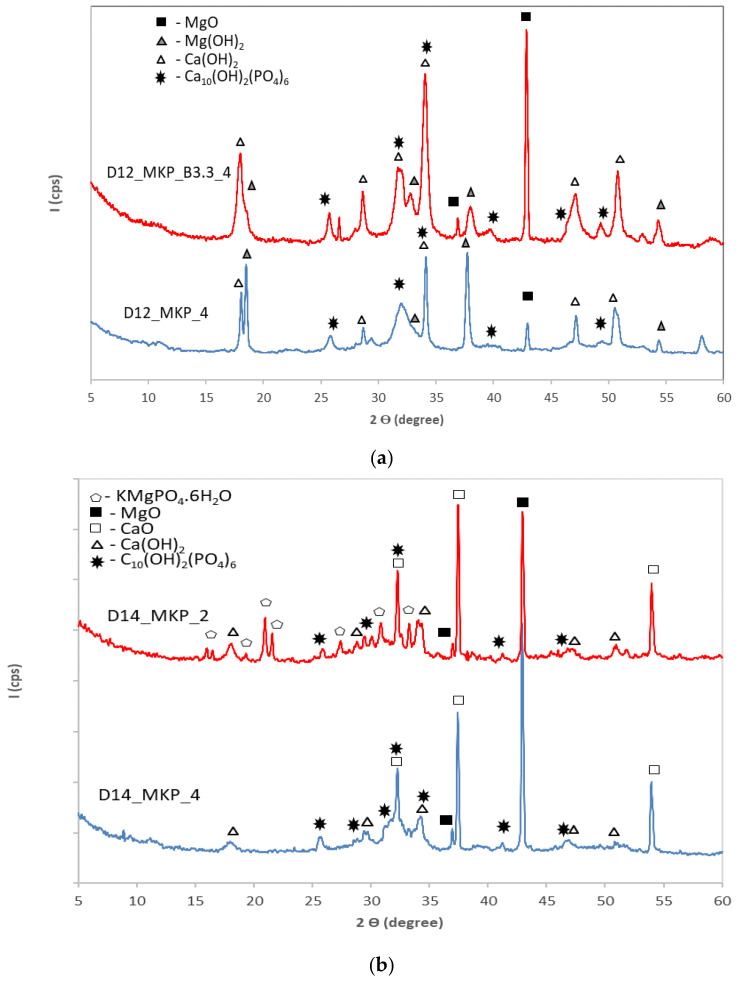
XRD patterns of the paste obtained by mixing water with potassium dihydrogen phosphate (with/without borax) and dolomite calcined for 3 h at (**a**) 1200 °C; (**b**) 1400 °C.

On the XRD patterns of cements based on dolomite with/without borax and MKP (Figure 5a) can be noticed the presence of calcium and magnesium hydroxides as well as the presence of hydroxyapatite (HAp)—which resulted in the reaction of calcium with phosphate, which was brought into the system by the potassium dihydrogen phosphate (MKP). The formation of HAp is also facilitated by the basicity of this system (the addition of MKP to the calcined dolomite + water mixture shifts the pH value at 8–9). 

Due to the high reactivity vs. water or MKP solution of dolomite calcinated at 1200 °C, phosphate cements based on dolomite calcined at 1400 °C were also obtained. 

Figure 5b shows the XRD patterns of phosphate cements based on dolomite calcined at 1400 °C prepared with different dosages of potassium dihydrogen phosphate (MKP). In the case of the specimens with a higher dosage of KH_2_PO_4_ (D_14__MKP_2) along with XRD peaks specific for magnesium oxide (004-0829), calcium oxide (082-1690), calcium hydroxide (084-1276) and HAp (084-1998) (which are also present on XRD patterns of D_14__MKP_4) appear also XRD peaks specific for K-struvite (KMgPO_4_.6H_2_O)(020-0685). Due to the higher reactivity of CaO as compared to MgO [24,27,28], HAp is the first reaction product formed in this system; if there are still available phosphate groups in the solution, K-struvite is formed by their reaction with magnesium. The presence of K-struvite contributes to the increase of mechanical strength [29,30]. 

These results are in correlation with the values of compressive strengths, which are presented in Table 3. The higher compressive strengths were assessed for the MPC based on calcined magnesite (M_MKP_4_B3.3) in which the main reaction product is K-struvite [30]. The specimens based on dolomite calcined at 1400 °C have recordable strengths only when the MgO/KH_2_PO_4_ ratio is 2, i.e., when K-struvite is detected in the hardened paste.

**Figure 6:**
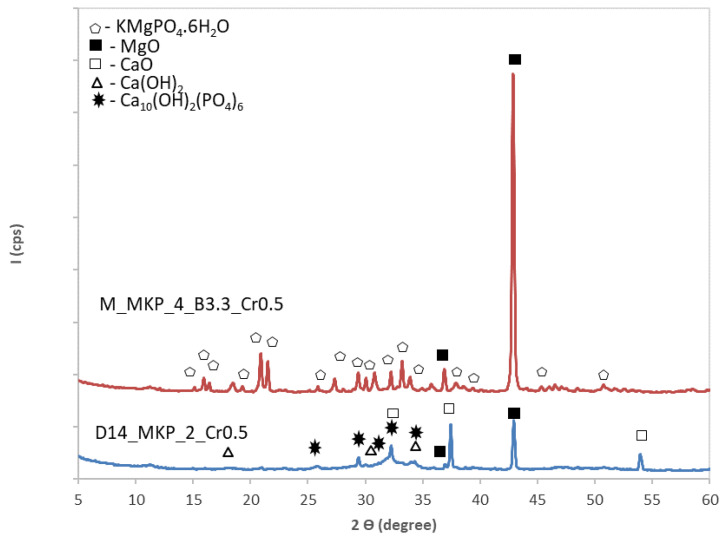
XRD patterns of the specimens based on calcined magnesite or dolomite calcined at 1400 °C with chromium waste corresponding to 0.5 wt % Cr.

In order to assess the influence of chromium waste on the composition of hardened phosphate cements, pastes with various amounts of waste were prepared (Table 1). The XRD patterns of the pastes based on calcined magnesite (M) and dolomite calcined at 1400 °C (D_14_) with a dosage of chromium waste corresponding to 0.5 wt % Cr are presented in Figure 6. 

For the paste based on magnesite (M_MKP_4_B3.3_Cr0.5), one can assess through this method the presence of MgO and K-struvite (KMgPO_4_.6H_2_O). The substitution of calcined magnesite with chromium waste determines an important decrease of the compressive strengths (Table 3), which could be due both to the smaller amount of K-struvite formed in the system (chromium waste substitute calcined magnesite and MKP) as well as the increase of water dosage (from 0.2 to 0.35—see Table 1) necessary to improve the workability of fresh paste.

For the specimen based on calcined dolomite (D_14_), the presence of chromium waste seems to inhibit the K-struvite formation (see also Figure 5b). This explains the decrease of compressive strengths values as compared with those recorded for specimen D_14__MKP_2, with the increase of Cr content (see Table 3). However, after 7 days of hardening, the compressive strength of specimens based on D_14_ (D_14__MKP_2 and D_14__MKP_2_Cr0.5) dramatically decrease, which is most probably due to a delayed hydration of free CaO and MgO. 

Therefore, in order to reduce the free lime content and to obtain magnesium oxide with an adequate reactivity, while keeping the same thermal treatment temperature of 1200 °C, a mixture of dolomite and quartz sand was thermally treated at this temperature for 1 h, based on the method proposed by Yu et al. [14].

**Figure 7:**
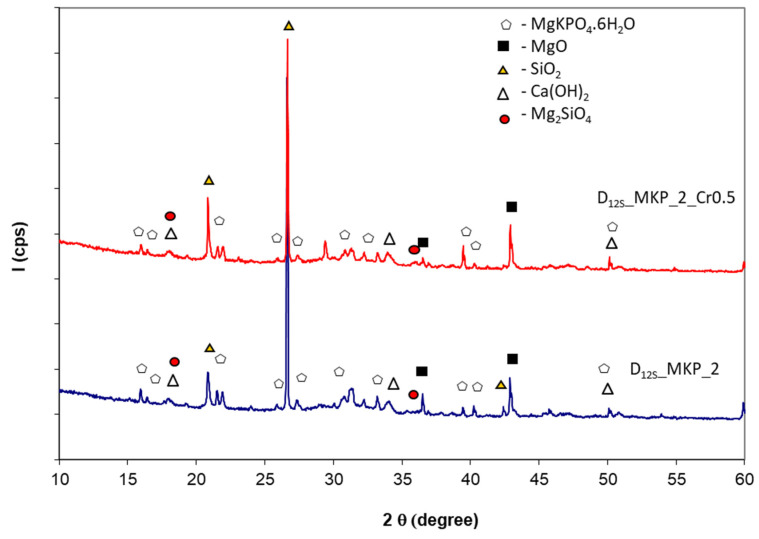
XRD patterns of the pastes obtained by mixing potassium dihydrogen phosphate and dolomite + sand calcined for 1 h at 1200 °C, with/without chromium waste.

The XRD pattens of the phosphate cement based on dolomite + sand calcined at 1200 °C—D_12S_ (Figure 7) show the presence of hydrates i.e., K-struvite and Ca(OH)_2_ along with MgO, SiO_2_, and Mg_2_SiO_4_ assessed in D_12S_ (see Figure 3). The presence of chromium waste does not change the nature of the reaction products (hydrates) assessed by this method (Figure 7).

**Figure 8:**
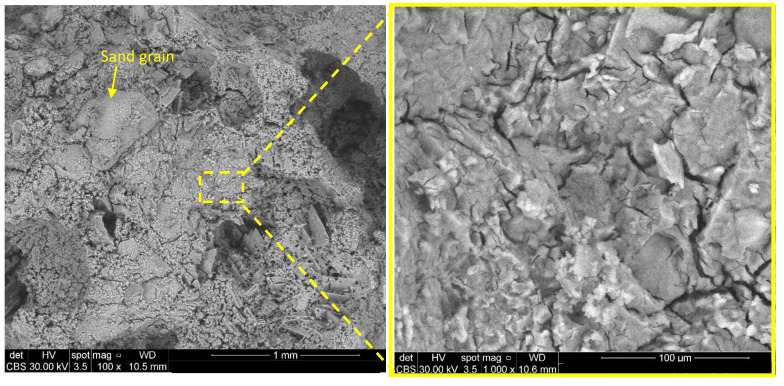
BSE images of D_12S__MKP_2.

In correlation with the above presented data, the compressive strengths of phosphate cements based on dolomite + sand calcined at 1200 °C (D_12S__MKP_2) are lower in comparison to the ones assessed for the phosphate cement based on magnesite (M_MKP_B3.3_4); however, these values steadily increase up to 28 days (Table 3). This compressive strength evolution can be related to the formation of K-struvite (assessed by XRD) and to the presence of sand grains, which act as aggregates (Figure 8).

**Figure 9:**
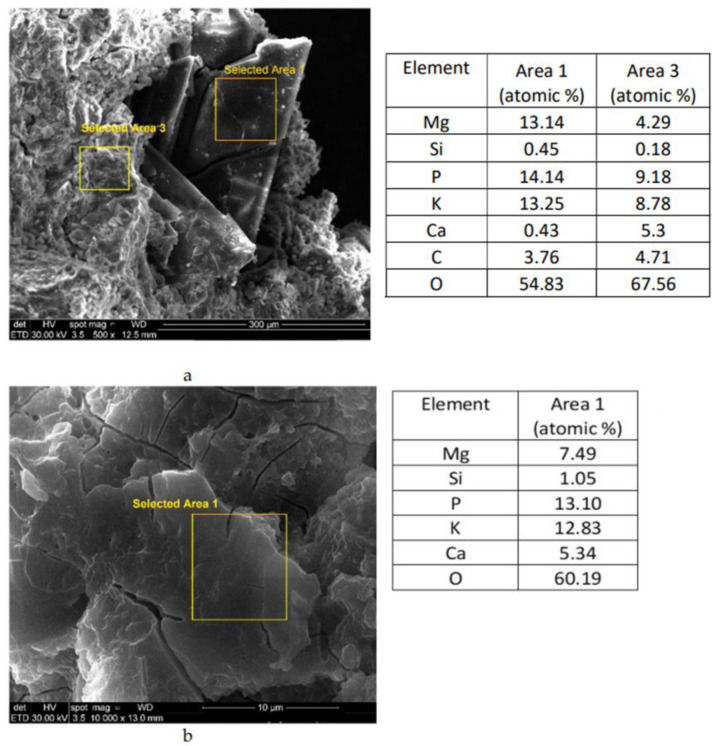
SEM images and EDX analyses of D_12S__MKP_2 cement paste at different magnifications: (**a**) ×500; (**b**) ×10,000.

Figure 9 presents the SEM images and elemental compositions assessed by EDX on various areas of D_12S__MKP_2 cement paste. As can be seen from Figure 9a, in area 1, the atomic ratio of K:Mg:P is 13.25:13.14:14.14 confirming the presence of K-struvite in these specimens; the elemental compositions in area 3 (Figure 9a) and area 1 (Figure 9b) show the presence of Ca together with Mg, K, and P, which suggest a complex composition of these hydrates [14]. 

For the cement paste based on D_12S_, the presence of chromium waste (in a dosage corresponding to 0.5 wt % Cr) determines a reduction of compressive strengths in comparison to the cement without Cr (D_12S__MKP_2); however, these values increase up to 28 days (Table 3). The decrease of compressive strengths can be explained by the lower amount of MgO (sand partially substitutes the dolomite) available in this system for the formation of K-struvite. 

**Figure 10:**
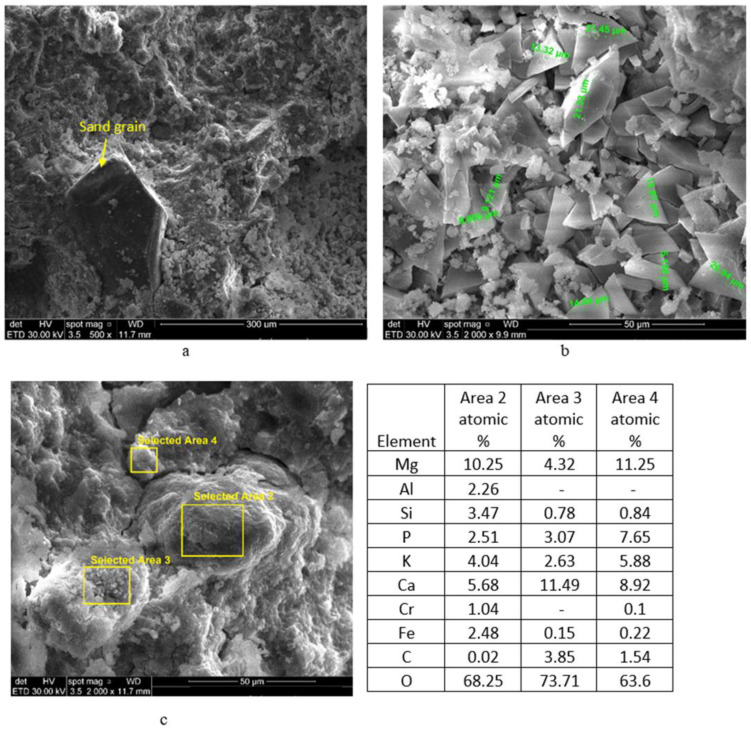
SEM images and EDX analyses of D_12S__MKP_2_Cr0.5 paste, at different magnifications: (**a**) ×500; (**b**) ×2000; (**c**) ×2000.

For a better understanding of the correlation between the morphology/composition and properties of D_12S__MKP_2_Cr0.5, SEM and EDX analyses were performed on this specimen (Figure 10). As it can be noticed, the sand grains are embedded in a continuous matrix (Figure 10a) in which are present plate-like and prismatic crystals intermixed with agglomerates of small grains (Figure 10b). The coherence of this matrix seems to be much lower in these specimens in comparison to the one without Cr (Figure 9), which can explain the lower mechanical strength values. 

The EDX analysis performed in three areas on SEM image presented in Figure 10c shows the presence of Cr, Al, and Fe (from waste [21]) mainly in area 2, which suggests the presence of a waste grain embedded in a layer (matrix) with Ca, K, and P content.

**Figure 11:**
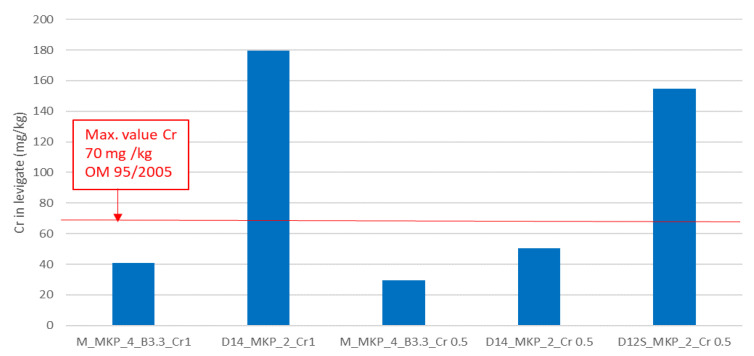
Cr content in levigates.

To assess the efficiency of the studied MPC and CMPC to immobilize Cr, a leaching test (described in SR EN 12457-4: 2003 [23]) was performed for phosphate cement pastes hardened for 28 days. The results are presented in Figure 11. 

In Figure 11, one can observe that the MPCs based on calcined magnesite are effective for the immobilization of chromium even for a high waste content (M_MKP_4_B3.3_Cr1); the Cr content determined in leachate is below the limit stipulated by the Romanian Ministerial Order OM 95/2009 [31] for both phosphate cement pastes based on calcined magnesite.

The good immobilization of Cr in the MPCs based on calcined magnesite (M) can be explained by the presence of K-struvite, which could play an important role [16,32]. Rouff [32] reported Cr adsorption or/and substitution in the struvite (NH_4_.H_2_PO_4_.6H_2_O), which precipitates from concentrate solutions of MgCl_2_.6H_2_O and (NH_4_)_2_HPO_4_, with Cr(NO_3_)_3_.9H_2_O or Na_2_CrO_4_ additions. 

For the phosphate cements based on calcined dolomite, only the one based on dolomite calcined at 1400 °C with a waste content corresponding to 0.5 wt % Cr (D_14__MKP_2_Cr0.5) fulfills the legal requirement. The reduced efficiency in immobilization of Cr in this type of cement can be due to the inhibition of K-struvite formation (suggested by XRD data—Figure 6). Nevertheless, the presence of hydroxyapatite (HAp) in this composition could contribute to the immobilization of Cr [33,34], explaining the low amount of chromium levigated in the CMPC with a lower chromium waste content (0.5 wt. %).

The high Cr content assessed in the levigate of the CMPC based on D_12S_ can be due to a low amount of K-struvite formed in this cement, due to partial substitution of dolomite with quartz sand. 

## 4. Conclusions

In this study, we obtained and studied the properties of magnesium phosphate and calcium magnesium phosphate cements based on calcined magnesite and calcined dolomite (at different temperatures) with/without sand addition and potassium dihydrogen phosphate.

The following conclusions can be drawn:

- The thermal treatment of dolomite at 1200 °C and 1400 °C leads to the decomposition of calcium magnesium carbonate into CaO and MgO. The increase in calcination temperature, from 1200 to 1400 °C reduces the reactivity of calcium and magnesium oxides vs. water or phosphate (MKP) solution; for the phosphate cements based on dolomite calcined at 1200 °C, an important increase in paste temperature during the setting and paste’s expansion was noticed due to the high reactivity of oxides (CaO and MgO); the increase of thermal treatment temperature at 1400 °C determines a decrease of the oxides’ reactivity, and for a higher KH_2_PO_4_ dosage (corresponding to D_14_/KH_2_PO_4_ = 2 weight ratio), the pastes have measurable compressive strength at early ages. Nevertheless, for all specimens based on dolomite calcined at 1400 °C, the compressive strengths dramatically decrease after 7 days of hardening, which is most probably due to a delayed hydration of CaO and MgO.
- The main compounds observed in hardened phosphate binders based on calcined dolomite were calcium and magnesium hydroxides; in the case of specimens with a lower dosage of MKP (corresponding to D_14_/MKP = 4 weight ratio)—along with Ca(OH)_2_ and Mg(OH)_2_, a new compound was detected by XRD—hydroxyapatite (HAp); HAp results from the reaction of calcium with phosphate, which is brought into the system by MKP. For a higher dosage of MKP (corresponding to D_14_/MKP = 2 weight ratio) on the XRD patterns, peaks specific for the K-struvite (KMgPO_4_.6H_2_O) compound are also present.
- In order to obtain a solid precursor for CMPC synthesis by the calcination of dolomite at relatively low temperature, a mixture of dolomite and quartz sand was thermally treated at 1200 °C for 1 h. The compressive strengths of resulting CMPC are lower in comparison with those assessed on phosphate cement based on calcined magnesite; however, they steadily increase up to 28 days. The lower values of compressive strengths assessed on these compositions are mainly due to the lower content of MgO available in this type of calcined dolomite (D_12S_) for the formation of K-struvite.
- The partial substitution of calcined magnesite and calcined dolomite with an industrial waste product with chromium content (corresponding to a Cr dosage of 0.5 wt % and 1 wt %) led to a significant decrease of compressive strength. In the case of MPC based on calcined magnesite, this decrease can be explained by the reduction of K-struvite amount due to the replacement of calcined magnesite and KH_2_PO_4_ with chromium waste, as well as by the increase of the water-to-solid ratio (necessary to obtain an adequate workability). For the CMPC based on dolomite calcined at 1400 °C (D_14_), the replacement of active components (D_14_ and KH_2_PO_4_) with chromium waste further inhibits the formation of K-struvite, and the compressive strengths decrease with the increase of the waste content. In the case of CMPC based on D_12S_, the lower compressive strengths assessed for specimen with chromium waste are also explained by the lower amount of K-struvite formed in this system (due to the decrease of MgO available for the reaction with KH_2_PO_4_).
- Phosphate cements based on calcined magnesite with a waste content corresponding to 1 and 0.5 wt % Cr can effectively reduce the Cr leaching for pastes cured for 28 days; in the case of phosphate cements based on calcined dolomite (D_14_), it was found that for a waste dosage corresponding to 0.5 wt % Cr, after 28 days of curing, the concentration of leached Cr is below the limit value imposed by the legislation currently in force. Nevertheless, considering the evolution of mechanical strength vs. time for this phosphate cement, it seems necessary to extend the evaluation of leached chromium for longer curing times (over 28 days). Although the CMPCs based on D_12S_ developed adequate compressive strengths even at longer hardening times (28 days), the amount of chromium leached exceeded the limit imposed by the current legislation most probably due to the decrease of K-struvite content. The chromium immobilization in this type of CMPC can be improved if the appropriate amount of D_12S_ (or chromium waste) is selected.

## Figures and Tables

**Table 1:** Compositions of phosphate cements based on calcined magnesite (M) and calcined dolomite (D) with/without chromium waste.

Sample	Calcined Magnesite (M) wt %	Calcined Dolomite (D) wt %	KH_2_PO_4_ (MKP) wt %	Borax * (B) wt %	Sand wt %	Cr Waste ** % wt %	M or D to MKPRatio (wt)	Water to Solid Ratio (wt)	Calcination Temperature (°C)
M_MKP_4_B3.3	80	-	20	3.3	-	-	4	0.2	1500
M_MKP_4_B3.3_Cr 1	50.4	-	12.6	3.3	-	37	4	0.35	1500
M_MKP_4_B3.3_Cr_0.5	64.8	-	16.2	3.3	-	19	4	0.35	1500
D_12__W	-	100	-	-	-	-	-	0.8	1200
D_12__MKP_4	-	80	20	-	-	-	4	0.67	1200
D_12__MKP_B3.3_4	-	80	20	3.3	-	-	4	0.67	1200
D_12__MKP_2.5	-	71.43	28.57	-	-	-	2.5	0.55	1200
D_12S__MKP_2	-	40.2	33	-	26.8	-	2	0.2	1200
D_14__MKP_4	-	80	20	-	-	-	4	0.3	1400
D_14__MKP_2	-	67	33		-	-	2	0.2	1400
D_14__MKP_2_Cr1	-	42	21	-	-	37	2	0.35	1400
D_14__MKP_2_Cr0.5	-	54	27	-	-	19	2	0.35	1400
D_12S__MKP_2_Cr0.5	-	32.4	27	-	21.6	19	2	0.2	1200

* Borax dosage was calculated with reference to calcined magnesite or calcined dolomite. ** Cr waste was dosed to bring in the system 0.5 wt % Cr and 1 wt % Cr; Cr waste substitutes the oxide + phosphate salt mixture.

**Table 2:** Maximum temperature (T_max_) and corresponding time (t_max_) for the studied binders.

Sample	T_max_ * (°C)	t_max_ ** (min)	Obs.
D_12__W	98	4	Expansion
D_12__MKP_4	90	26	Expansion
D_12__MKP_2.5	90	10	Expansion

* T_max_—maximum temperature (°C) of paste assessed after the mixing of precursors; ** t_max_—time (minutes) corresponding to Tmax.

**Table 3:** Compressive strengths versus time. Influence of Cr waste presence.

Specimens	Compressive Strength (MPa)			
	1 Day	3 Days	7 Days	28 Days
M_MKP_4_B3.3	16.87	21.62	26.5	27.1
M_MKP_4_B3.3_Cr0.5	0.6	1	1.15	1.5
M_MKP_4_B3.3_Cr1	0	0	0	0
D_14__MKP_4	0	0	0	0
D_14__MKP_2	7.1	10.8	12.2	0
D_14__MKP_2_Cr0.5	1.5	1.7	2	0
D_14__MKP_2_Cr1	0	0	0	0
D_12S__MKP_2	2.8	6.3	7.2	9.2
D_12S__MKP_2_Cr0.5	0	3.5	5.4	5.4

## Data Availability

Not applicable.
